# High-Intensity Sports Participation Induces Itch-Associated Sensitive Skin

**DOI:** 10.1007/s43657-025-00255-4

**Published:** 2026-04-09

**Authors:** Yanqing Liu, Fushuo Li, Xinyuan Zhang, Chungan Liao, Ritsuko Yamazaki, Ichiki Fukuda, Jing Liu, Weilin Pu, Yiyu Wang, Wei Liu, Jiucun Wang, Yanyun Ma

**Affiliations:** 1 State Key Laboratory of Genetic Engineering, School of Life Science, and Human Phenome Institute, Zhangjiang Fudan International Innovation Center, Fudan University, Shanghai, 200438 P.R. China; 2 Kao (China) R&D Center Co.,Ltd, Shanghai, 200241 P.R. China; 3 Shanghai Skinshield Clinical Testing and Technological Research Ltd, Shanghai, 200433 P.R. China; 4 Greater Bay Area Institute of Precision Medicine (Guangzhou), School of Life Sciences, Fudan University, Guangzhou, 511462 P.R. China; 5 Department of Dermatology, Air Force General Hospital, Beijing, 100142 P.R. China; 6 Academy for Engineering & Technology, Fudan University, Shanghai, 200082 P.R. China; 7 Research Unit of Dissecting the Population Genetics and Developing New Technologies for Treatment and Prevention of Skin Phenotypes and Dermatological Diseases (2019RU058), Chinese Academy of Medical Sciences, Beijing, 100700 P.R. China

As the body’s outermost organ, the skin is constantly exposed to environmental irritants and responds upon contact. When skin functions are compromised, environmental allergens can penetrate the body, potentially leading to dermatological conditions such as dermatitis and psoriasis (Proksch et al. 2008). Although previous literature has documented the positive effects of physical activity on physical and mental health (Galper et al. 2006), emerging evidence indicates that sports participation can also be associated with various skin issues.

It was reported that cutaneous diseases account for 20.9% of college sports-related conditions, with athletes at higher risk of developing skin issues (Pujalte et al. 2022). The symptoms included talon noir, swimmer’s itch, and *acne mechanica* (Pharis et al. 1997). Most studies on sports-related skin conditions focused on allergic reactions (Adams 2002), bacterial infections (Kermani et al. 2019), and environmental skin damage (De Luca et al. 2012), which generally arise from direct contact. Yet, the intrinsic effects of sports participation on facial skin, particularly itch, remain underelucidated. To address this gap, we conducted two waves of surveys to qualitatively and quantitatively explore the relationship between physical activity and itching conditions. Additionally, we aimed to develop a practical diagnostic indicator for sport-induced sensitive skin (SSS) through physical activity interventions in healthy volunteers.

The sport discontinuation rate was 7.23%, of which 65.52% cited sport-induced skin discomfort as the primary reason (Fig. 1a−1b). Among active participants, 61.56% reported skin discomfort during sports (Fig. 1c). Notably, significant associations were observed between skin sensitivity and reported discomfort (*p* < 0.001, Table 1), which link exercise and cutaneous symptoms. Itch was the predominant symptom during (40.09%) and after (36.11%) physical activity (Fig. 1d−1e). Logistic regression revealed that anaerobic sports significantly increased the likelihood of experiencing itch (OR = 2.78, *p* < 0.001) (Table 2). Additionally, high-intensity exercise was associated with a higher risk of itch manifestation (OR = 6.01, *p* < 0.05), whereas sport duration was not.

**Table 1:** A 2 × 2 contingency table of skin discomfort during sport and skin sensitivity

	Skin Sensitivity		*p-*value
	Insensitive	Sensitive	
**Skin Discomfort During Sport**			< 0.001
**No**	90	73	
**Yes**	31	207	

Note: Chi-square test of association between categorical variables

**Table 2:** Logistic regression model for associations between itch and sport type, duration, and intensity

	Itch manifestation
	Odds Ratio (95%CI)
Sport Type	
Aerobic	0.73 (0.29 ~ 1.80)
Anaerobic	2.78 *** (1.52 ~ 5.10)
**Sport Duration**	
> 1.5 h	1.57 (0.62 ~ 3.98)
**Sport Intensity**	
Moderate (100-140 bpm)	1.13 (0.62 ~ 2.09)
High (> 140 bpm)	6.01 * (1.24 ~ 29.14)

Note: *** *p* < 0.001, ** *p* < 0.01, * *p* < 0.05

SSS was defined by the gold-standard lactic acid sting test (LAST) score, which increased significantly from < 3 to ≥ 3 after sports interventions (26.05%, *p* < 0.001). Overall, 55.81% of participants met the SSS criteria (Fig. 2a). The SSS group had significantly higher mean itch numeric rating scale (NRS) scores during (*p* < 0.001) and after (*p* < 0.001) exercise than the non-sport-induced sensitive skin (NSSS) group (Fig. 2b). The receiver operating characteristic analysis (ROC) identified itch NRS ≥ 4 as a supportive diagnostic criterion (Fig. 2c).

The SSS group exhibited more severe skin symptoms during physical activity, including a significantly greater increase in itch NRS scores (*p* < 0.001), self-reported itch (*p* < 0.001), sting (*p* < 0.001), and erythema (*p* < 0.05), which persisted after exercise, highlighting the prolonged impact of SSS on skin health (Fig. 2d−2g).

Our findings indicate that higher exercise intensity significantly increases the likelihood of sport-induced itch, particularly in anaerobic sports. The high prevalence of sport-induced skin sensitivity underscores the importance of addressing sport-induced skin responses.

In this study, we propose an itch NRS score ≥ 4 as a user-friendly indicator for diagnosing SSS. This method aligns with LAST-based assessments while avoiding irritant application (Supplementary Fig. 4). Moreover, individuals with a history of dermatological conditions are more prone to sport-induced skin reactions. Our study population had minimal preexisting skin issues, with only 89 out of 401 reporting a history of *cholinergicurticaria* and none reporting medical skin conditions at the validation phase, minimizing the likelihood that our findings were confounded by underlying dermatological disorders.

This study possesses several limitations. First, we primarily focused on Chinese young adults due to their higher compliance rates for feasible data collection. Thus, the generalizability of the observed associations and the proposed alternative SSS indicator to broader populations may be restricted. Second, based on previous findings on skin sensitivity symptoms associated with exercise (Simmons et al. 2011; Takahagi et al. 2022), we hypothesized that SSS may be a short-term, transient phenomenon. Thus, the sporting interventions in our study lasted only one hour. As a result, the long-term effects of repeated physical exercise remain unexplored. Future investigations on longitudinal monitoring of athletes engaged in sustained high-intensity training are therefore warranted.

SSS is prevalent among physically active Chinese individuals, with high-intensity exercise as a key factor. The proposed itch NRS ≥ 4 diagnostic approach offers a practical alternative to traditional assessments. Emerging evidence (Ying 2023; Zhao et al. 2024) highlights the need for further research on skin phenomics and appendages to identify novel biomarkers and personalized therapies. Lastly, validating the itch NRS diagnosis is crucial for developing targeted interventions for SSS.

**Fig. 1:**
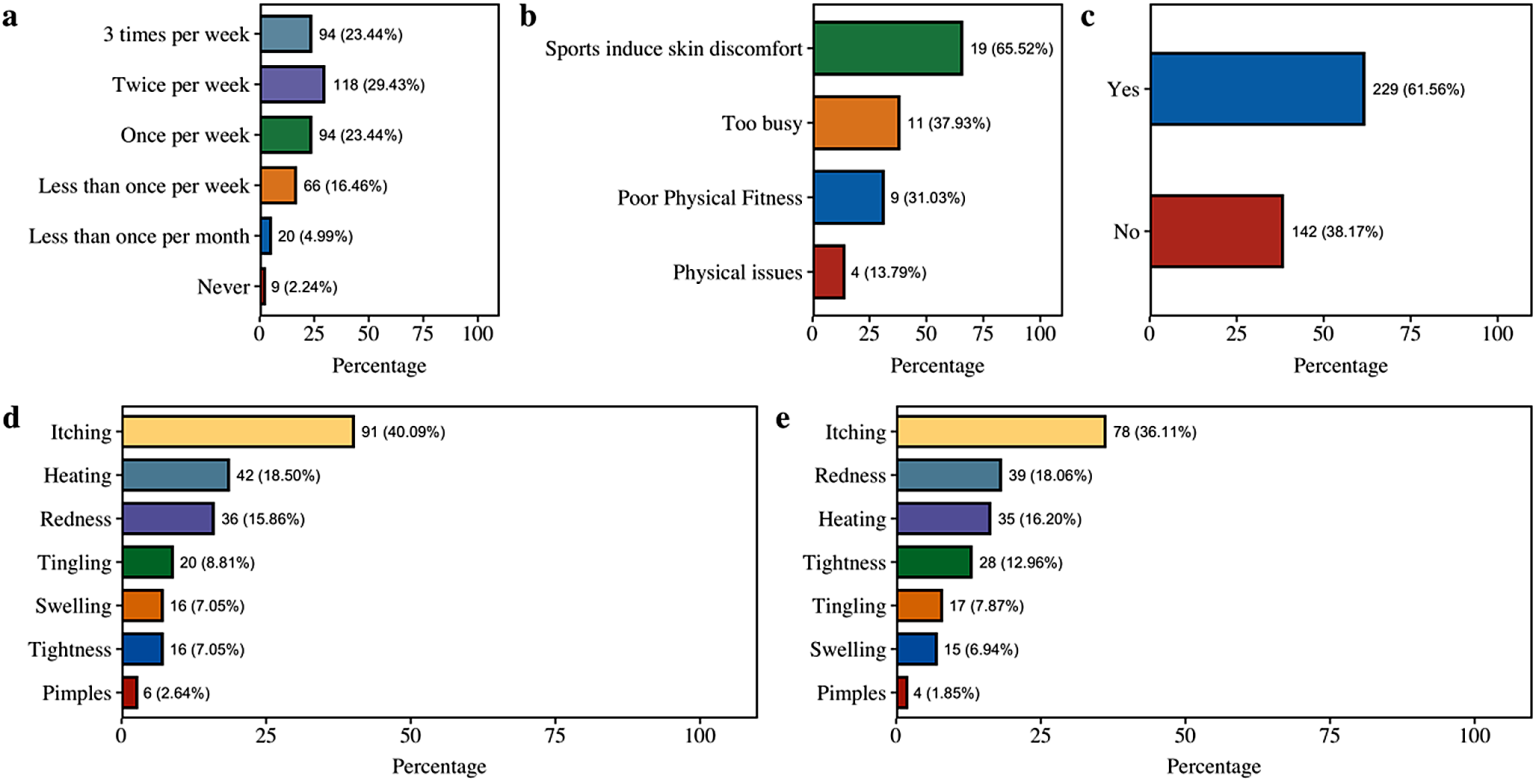
Sporting habits and related skin discomfort in surveyed participants. 1a. “Do you engage in sport activities?”; 1b. “If not in 1a, what are the reasons for not engaging in sport?”; 1c. Proportion of skin discomfort in sporting population; 1d−1e. Main types of skin discomfort during and after sporting activities

**Fig. 2:**
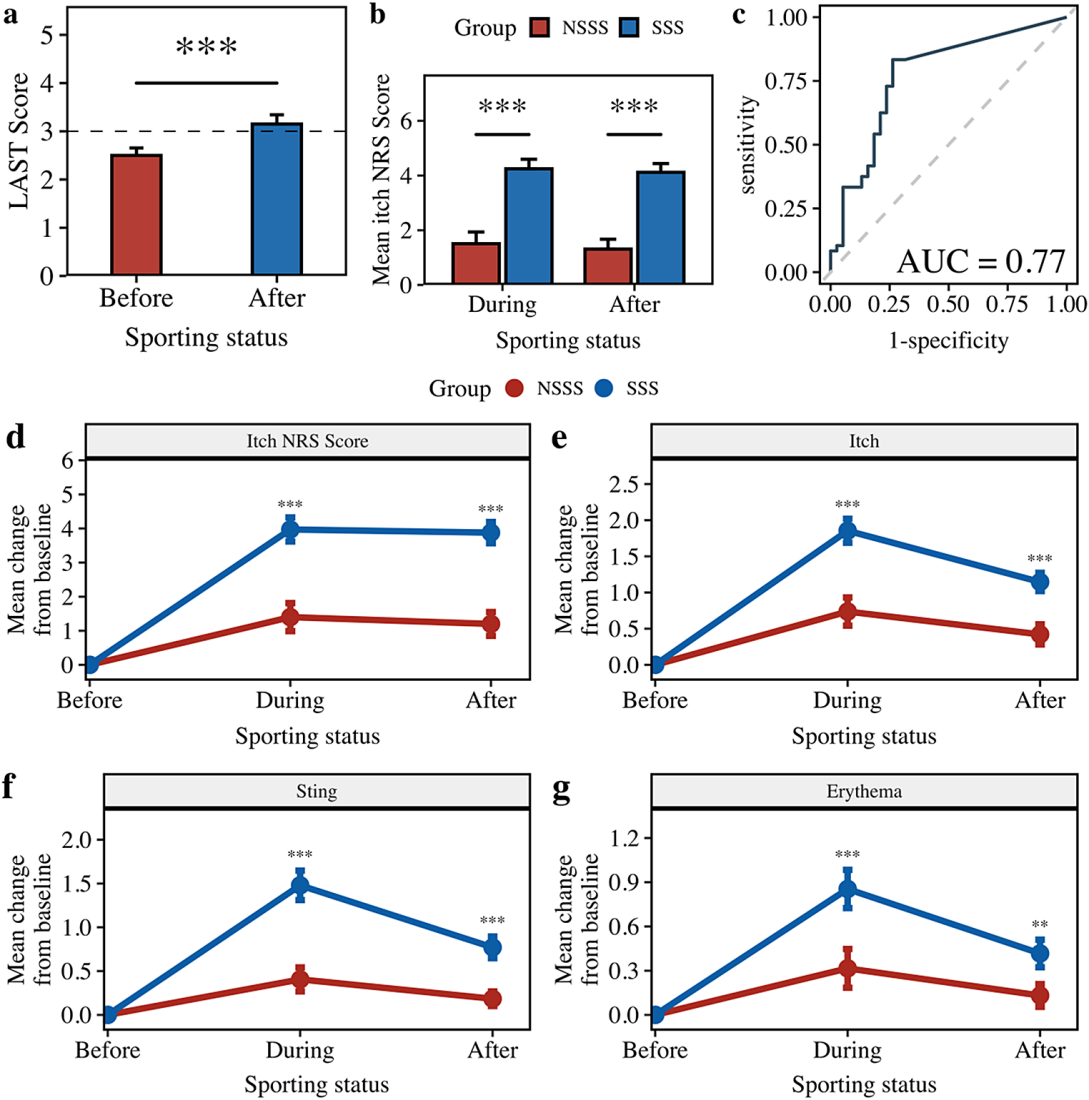
Defining sport-induced sensitivity (SSS) and the use of mean NRS score as an indicator for SSS. 2a. Change in the LAST score over the course of sporting activities; 2b. Mean itch NRS score over the course of sporting acitivities for SSS and NSSS groups; 2c. ROC curve for identification of optimal mean itch NRS threshold to support SSS diagnosis. Area under the curve (AUC) = 0.77 represents satisfactory diagnostic performance of the itch NRS scores; 2d − 2g. Mean change in subjects’ self-assessed skin condition over the course of sporting activities. *** *p* < 0.001, ** *p* < 0.01, * *p* < 0.05

## Electronic Supplementary Material

Below is the link to the electronic supplementary material.

Supplementary Material 1

## Data Availability

The data supporting the findings of this study are available upon request from the corresponding author, YYM. The data are not publicly available due to confidentiality concerns, as they contain sensitive information that could compromise the privacy of the research participants.

## Abbreviations

SSS: Sport-induced sensitive skin
OR: Odds ratio
CI: Confidence interval
LAST: Lactic acid sting test
NRS: Numeric rating scale
AUC: Area under the curve
ROC: Receiver operating characteristic
