# Supplementary material for: High-Intensity Sports Participation Induces Itch-Associated Sensitive Skin
Source: Phenomics. 2026 Apr 9;6(1):82–5. doi: 10.1007/s43657-025-00255-4 (PMC13226766; doi:10.1007/s43657-025-00255-4)
Supplement: Supplementary file 1 — Supplementary Material 1 [file 43657_2025_255_MOESM1_ESM.docx]

**Supplementary Materials**

**Methods**

*Participant Recruitment and Flow Chart of Sample Exclusion*

Supplementary Fig 1a illustrated the flow of the designed online questionnaires. Initially, 401 subjects were recruited. If a subject’s response fell into the “non-sporting” category, subsequent sports- and itch-related questions were skipped, excluding 29 respondents and leaving 371 in the sporting cohort. For the sporting cohort, questions were categorized into “during” and “after” sporting activities, with identical or similar questions for each category. A total of 227 and 216 individuals were included in the “during” and “after” sections, respectively. Subjects who reported discomfort during or after sports that was not related to itching were further excluded, resulting in a final sample of 96 subjects whose data were used to construct logistic regression models. In the validation phase, the exclusion criteria included:

1. Presence of diseases or physical discomfort, including but not limited to immune or systemic diseases such as diabetes, hepatitis, hypothyroidism, or active skin conditions such as eczema, psoriasis, vitiligo, acne, seborrheic dermatitis, or any other condition deemed by the PI or a medically qualified appointee to potentially affect the study results.
2. Currently using topical or oral medications that may affect the study, including: a) Use of immunosuppressants, steroidal and non-steroidal anti-inflammatory drugs, or prescribed hormonal medications (including hormone therapy, insulin, etc.) within the past three months; b) Use of antibiotics or hormones within the past month.
3. Presence of facial cuts, abrasions, or other skin conditions deemed by the PI to potentially affect assessments and instrument testing.
4. Currently participating in any other clinical study or having participated in any clinical study involving the test area within the past three months.
5. Use of any cosmetic products on the day of the trial, including but not limited to skincare products, sunscreen, foundation, or makeup.

In total, 16 out of 102 subjects were disqualified, leaving 86 participants who received the sporting interventions (Supplementary Fig. 1b).

*Sporting-related and Itch-related Features*

Attributes related to sporting habits were assessed, including the type of sport, duration of sporting activity, and intensity of sporting activity. These three features were chosen to encompass various dimensions of sporting behavior. Parameters associated with itching were also investigated, comprising: (1) duration of itch, and (2) intensity of itch. The collected results of the above features are displayed as supplementary materials (Supplementary Fig. 2).

*Lactic Acid Sting Test*

At validation phase, participants were tested their skin sensitivity using the “gold standard”, LAST score. Fifty microliter of 10% lactic acid solution was prepared with ultra-pure water. The solution was randomly applied to one side of subject’s nasolabial fold area. The sum of scores at 2.5 min and 5 min for each subject yields one’s total LAST score, and a score of ≥ 3 was defined as sensitive skin.

*Itch NRS Score and Other Clinical Measurements*

Each subject’s itch level was quantified using the itch NRS throughout the course of sporting activity. The itch NRS score ranges from 0 to 10, with 0 being no itch at all, and 10 being extreme itch. In the present study, the itch NRS scoring was established as an accurate indicator for identification of SSS. Other measurements such as self-assessed scores were also acquired throughout the participants’ exercises.

*Statistical Analysis*

Data analyses were performed with R version 4.3.1. To assess associations between categorical variables at survey phase, logistic regression models were employed to derive odds ratios (OR) for evaluating the effect sizes of the associations.

At clinical validation phase, comparisons of means between groups at each time point were performed with independent the Wilcoxon rank-sum test, and the paired Wilcoxon rank-sum test was used for comparisons of change in measurements before and after sporting intervention. A significance level of *p* < 0.05 was applied throughout the present study unless otherwise stated.

In validation of itch NRS score threshold as an accurate SSS indicator, the ROC curve was drawn and used to identify optimal cut-off value.

**Supplementary Results**

*Subjects’ Demographics Characteristics*

Subjects' characteristics are summarized in Supplementary Table 1. Gender distributions at two phases were balanced, but the surveyed population was slightly older than the validation cohort. The prevalence of skin sensitivity was 69.83% in the surveyed population versus 55.81% in the validation cohort.

*Regional differences of Sports-induced Itch*

Regional differences in itch intensity were assessed by categorizing body sites into scalp, neck and ears, torso, limbs, and multiple regions. While subjects with itch symptoms in multiple sites had higher average itch NRS scores, no significant regional difference was observed in overall itch intensity (*p* = 0.171, Supplementary Fig. 3a). However, the onset of itch varied across anatomical regions, with the torso experiencing symptoms earliest (Log-rank *p* = 0.041, Supplementary Fig. 3b). These findings suggest that while itch severity remains similar across body areas, symptom onset may be site-dependent.

*Validation of the cross-sectional findings*

To validate the cross-sectional findings, we conducted a clinical study in which subjects underwent controlled sporting interventions. Since exercise intensity lacks a universal gold-standard measure, we utilized sweat frequency as a surrogate marker (Alig & Muntwyler, 2002). Our results reaffirmed that increased exercise intensity was significantly associated with SSS (OR = 2.88, 95%CI = [1.47 – 6.19]).

**Supplementary Figures**

**Supplementary Fig.** 1. Participants recruitment and flow charts of sample exclusion. 1a: survey phase samples and; 1b: clinical phase samples.


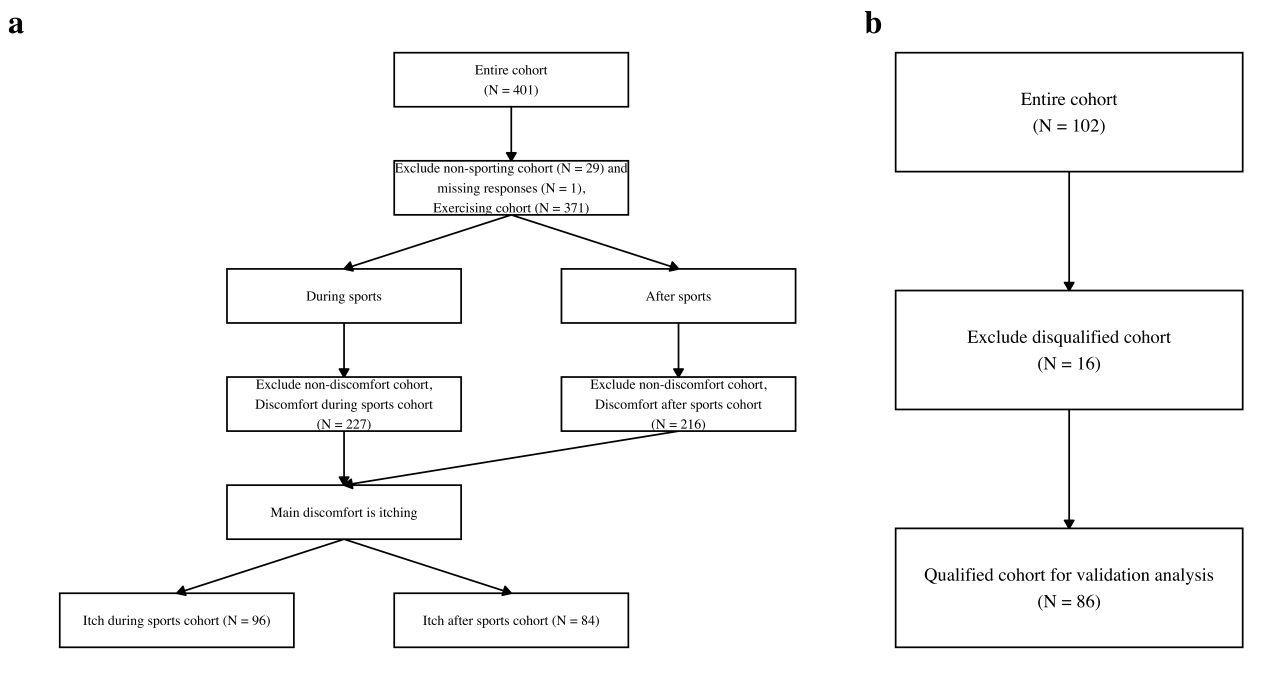


**Supplementary Fig.** 2. Sport- and itch-related features. 2a. Sporting intensity; 2b. Sporting duration; 2c. Itch intensity; 2d. Itch duration.


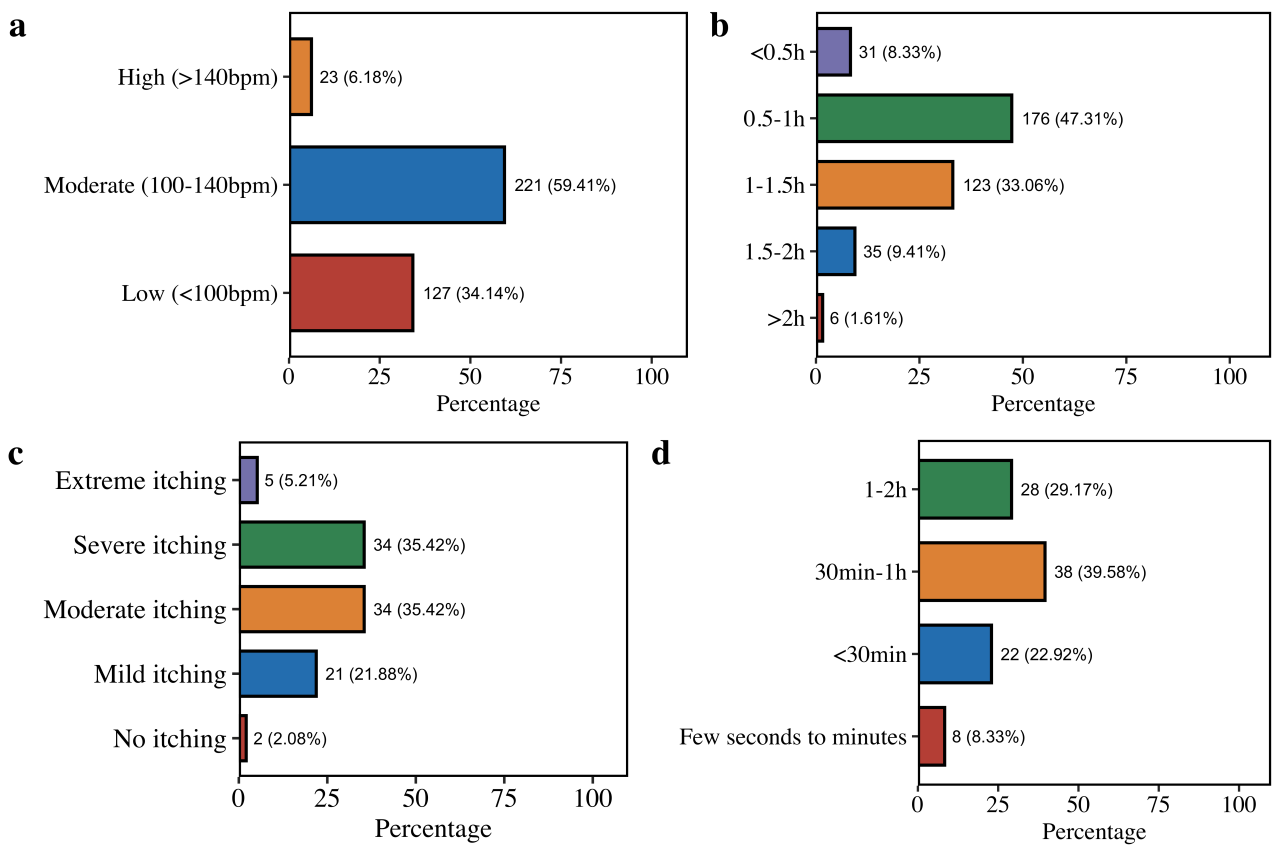


**Supplementary Fig.** 3. Itch conditions at different body sites. 3a. Mean itch NRS score over the course of exercise; 3b. Survival analysis on time to itch symptom at each body site.


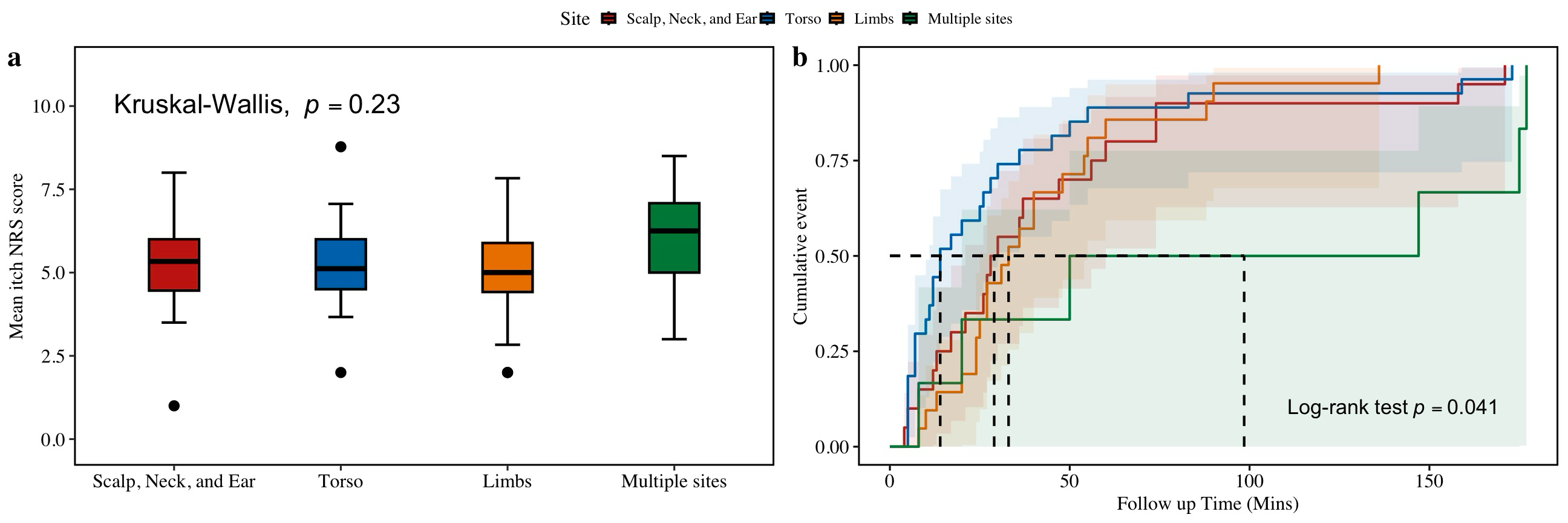


**Supplementary Fig.** 4. Mean change in subjects’ self-assessed skin condition over the course of sporting activities, groups classified by NRS ≥ 4. *** *p* < 0.001, ** *p* < 0.01, * *p* < 0.05, the Wilcoxon rank-sum test was used.
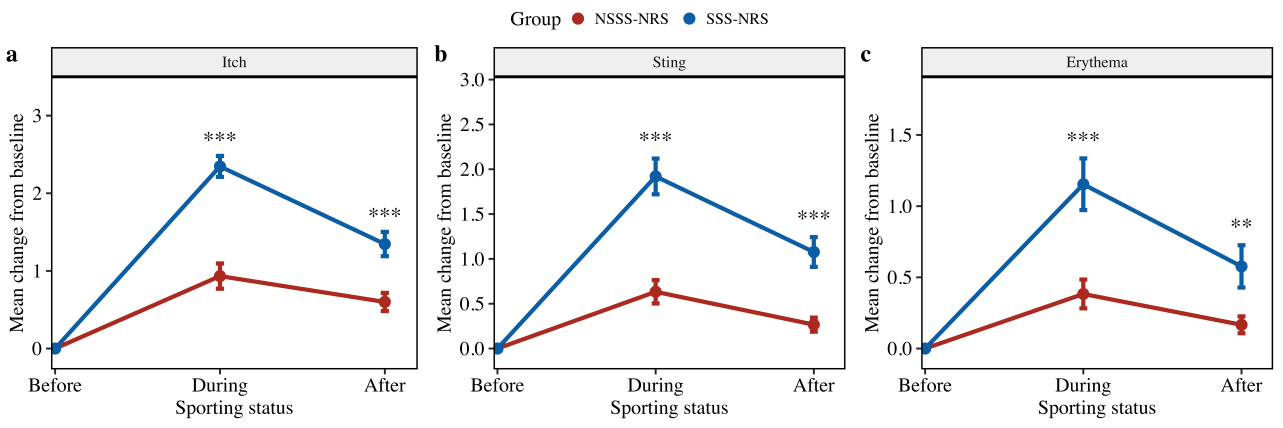


**Supplementary Tables**

Supplementary Table 1. Baseline demographics of the participants.

| **Characteristic** | **Survey phase (N = 401)** | **Validation phase (N = 86)** |
| --- | --- | --- |
| **Age (years)** |  | 24.07 ± 3.84 |
| **Age group** |  |  |
| <18 | 2 (0.50%) |  |
| 18-24 | 86 (21.45%) | 48 (55.81%) |
| 25-30 | 145 (36.16%) | 36 (41.86%) |
| 31-40 | 138 (34.41%) | 2 (2.33%) |
| 41-50 | 29 (7.23%) |  |
| 51-60 | 1 (0.25%) |  |
| **Gender** |  |  |
| Female | 204 (50.87%) | 44 (51.16%) |
| Male | 197 (49.13%) | 42 (48.84%) |
| **BMI (kg/m²)** |  | 23.73 ± 5.58 |
| **Skin type** |  |  |
| Dry | 62 (15.46%) | 44 (51.16%) |
| Dry Mixed | 49 (12.22%) |  |
| Neutral | 105 (26.18%) |  |
| Oily | 119 (29.68%) | 42 (48.84%) |
| Oily Mixed | 66 (16.46%) |  |
| **Skin sensitivity** |  |  |
| No | 121 (30.17%) | 38 (44.19%) |
| Yes | 280 (69.83%) | 48 (55.81%) |
| **Note:** Mean ± standard deviations were calculated for continuous variables, and categorical variables were reported with frequencies and percentages. | | |
